# Arthroscopic incidence of lateral meniscal root avulsion in patients with anterior cruciate ligament injury

**DOI:** 10.1186/s10195-021-00591-x

**Published:** 2021-07-18

**Authors:** Riccardo Ciatti, Armando Gabrielli, Germando Iannella, Pier Paolo Mariani

**Affiliations:** Casa Di Cura Villa Stuart. Via Trionfale, Rome, 5952 00135 Italy

**Keywords:** Meniscal root, ACL injury, Lateral meniscus, Incidence, Knee

## Abstract

**Background:**

To arthroscopically evaluate the incidence of lateral meniscal root avulsion (LMRA) and associated intra-articular injuries in patients undergoing anterior cruciate ligament (ACL) reconstruction.

**Materials and Methods:**

From April 2014 to March 2017, 532 consecutive patients were diagnosed as having an ACL injury and underwent arthroscopic ACL reconstruction. The diagnosis of LMRA was made arthroscopically. The effects of gender, activity, grade of laxity, time from injury, and concomitant meniscal lesions were analyzed.

**Results:**

Among 532 patients, 497 (93.4%) underwent primary ACL reconstruction and 35 (6.5%) underwent revision procedures. 383 were acute or subacute injuries (less than 6 months from injury to surgery) and 149 chronic (more than 6 months). Average age was 30.4 years (DS: ± 11.04); there were 422 (79.3%) males and 110 (20.6%) females. A LMRA associated with the ACL injury was detected in 72 cases (13.5%), with a significant prevalence observed in males (${\chi ^2}$ = 4.65; *P* = 0.031, statistically significant). In the 149 patients with a chronic injury, 27 patients had LMRA (18.1%), while 45 of the 383 patients with an acute or subacute injury had LMRA (11.7%). There was a tendency, albeit not significant (${\chi ^2}$ = 3.721; *P* = 0.054), for the prevalence to increase with time since the initial ACL injury. LMRA was significantly associated (${\chi ^2}$ = 7.81; *P* = 0.006) with a meniscocapsular tear of the posterior horn of the medial meniscus (ramp lesion). No other significant associations, such as with severity of A-P translation (as measured by KT-2000) or activity level, were detected.

**Conclusion:**

LMRA is a relatively common injury associated with both acute and chronic ACL tears. A relatively high incidence in cases of chronic ACL insufficiency suggests that LMRAs do not heal spontaneously or that they may appear with time, even when absent at the time of the initial injury.

**Level of evidence:**

Level III, cross-sectional study.

## Introduction

Lateral meniscal root avulsion (LMRA) is a relatively common lesion found in association with anterior cruciate ligament (ACL) rupture [1–3, 9]. Most papers about LMRA mainly focus on repair techniques and clinical outcomes, and data regarding its incidence and its associations with other intraarticular lesions and the timing of ligamentous injury surgery (acute or chronic) are scarce [1–3]. In the radiological literature [4, 5], the reported incidence of an ACL tear varies from 8 to 9.8%. In the orthopedic literature [2, 6, 7], its reported incidence is more variable, ranging from 6.7 to 12.4%. This discrepancy may be due to difficulties in radiologically diagnosing LMRA, as the MRI signs are not unequivocal, and various degrees of extrusion of the lateral meniscus are usually seen in association with ACL tears, even with an intact meniscal root [8]. Moreover, the term “meniscal root tear” can be used to refer to different types of meniscal root lesions, leading to some confusion. Currently, the most widely used classification of meniscal root lesions is that of LaPrade, which divides LMRAs into five types: partial and stable root tears (type 1), complete radial tears within 9 mm of the bony root attachment (type 2), bucket-handle tears with complete root detachment (type 3), complex oblique or longitudinal tears with complete root detachment (type 4), and bony avulsion fractures of the root attachment (type 5) [9]. All these lesions are associated with ACL rupture and probably have different traumatic mechanisms and outcomes.

The aim of this study was to prospectively determine the prevalence of LMRA and its associations with other intraarticular injuries and relevant risk factors. Our hypothesis was that LMRA is distinct from other lateral meniscal posterior horn tears, with a higher incidence in patients with an acute or chronic ACL tear, and that the spontaneous healing that has been reported to occur for other posterior lateral meniscal tears found in association with ACL rupture (partial thickness tears or complete oblique or radial tears of the posterior horn without detachment from the root) may not occur for LMRAs, as suggested by the presence of this lesion in patients with chronic ACL deficiency.

## Materials and methods

We prospectively evaluated 532 consecutive patients who underwent ACL arthroscopic reconstruction from April 2014 to March 2017. The inclusion criteria were: ACL reconstruction or repair and ACL revision surgery. Exclusion criteria were: previous meniscal surgery, tibial ACL avulsion, and associated PCL or peripheral injuries requiring surgery (Table 1). ACL reconstruction was performed by a transtibial technique using the patellar tendon or hamstring tendons, depending on the specific patient. Graft choice had no influence on the epidemiological purpose of this paper.

**Table 1:** Patient recruitment criteria for LMRA lesions

Inclusion criteria	Exclusion criteria
ACL injury treated with ACLR	Previous meniscal surgery
ACL revision surgery	ACL tibial avulsion fracture
	Associated PCL injury or peripheral injury requiring surgical repair or reconstruction

Preoperatively, all patients underwent standardized clinical, instrumental (KT-2000™), and MRI evaluations. The final diagnosis of ACL rupture and associated lesions—if present—was made arthroscopically, in concomitance with the ACL reconstruction procedure. The series consisted of 497 (93.43%) primary reconstructions and 35 (6.57%) revision procedures, with an average patient age of 30.4 years (DS: ± 11.04). We considered two groups of patients regarding the timing of surgery: acute/subacute (less than 6 months from injury to surgery) and chronic (more than 6 months). The prevalence of LMRA was calculated for each. We only considered LMRAs that were true tibial avulsions, not radial or longitudinal tears within 1 cm from the root.

The following data were collected: (1) gender; (2) time from injury to surgery; (3) sporting activity (Tegner); (4) anterior tibial translation as measured via KT-2000; and (5) associated medial and lateral meniscus injuries identified at arthroscopic evaluation. Three levels of sporting activity were distinguished according to the Tegner score: 10–9; 7–5; < 5. Anterior tibial translations were divided into four groups described by the IKDC classification: A (from 0 to 3 mm), B (from 3 to 5 mm); C (from 6 to 10 mm); D (> 10 mm). Associated meniscal injuries were described in terms of the tear morphology as a flap, radial, bucket handle or longitudinal, or a horizontal tear or a ramp lesion. This study received institutional review board approval.

### Statistical analysis

Statistical analysis was performed using the software package IBM SPSS Statistics 20 for Windows (release 21.0.0, IBM Corporation, 2012). The descriptive indicators calculated were absolute frequencies and percentages. The chi-squared test was used to test the significance of associations between qualitative variables, along with the Yates continuity correction because the variables in association were both dichothomous in every test. If the expected count was less than five, the significance from Fisher’s exact test was used. A two-sided significance of 0.05 was used for all analyses.

## Results

### Arthroscopic prevalence of lateral meniscal root avulsion

Among the 532 patients with ACL injuries, there were 72 cases (13.5%) of confirmed LMRA. LMRA was present in 63 (12.6%) primary reconstruction cases and in 9 (25.7%) cases involving revision procedures that is not statistically significant (${\chi ^2}$: 0.878; *P*: n.s) (Table 2). There were 422 (79.3%) males and 110 (20.6%) females. The prevalence of LMRA in male patients (*n*: 422) was 15.2% (*n* = 64), while the prevalence in female patients (*n*: 110) was 7.3% (*n* = 8). This difference in prevalence was statistically significant (${\chi ^2}$: 4.65;* P*: 0.031) (Table 3).

**Table 2:** Prevalence of LMRA in primary reconstructions and revisions

Procedure	No. of patients	Total no. of LMRAs	Prevalence
Primary ACL reconstruction	497	63	12.6%
ACL revision surgery	35	9	25.7%

**Table 3:** Prevalence of LMRA in male and female patients

Gender	No. of patients	Total no. of LMRAs	Prevalence
Male	422	64	15.2%
Female	110	8	7.3%

### Time from injury

In our series, 383 were acute or subacute injuries and 149 were chronic ACL-deficient knees. In the first group, 45 patients had LMRA (11.7%); in the latter group, 27 patients had LMRA (18.1%). The prevalence was near the significant level, 0.05 (${\chi ^2}$: 3.721; *P*: 0.054) (Table 4).

**Table 4:** Prevalence of LMRA in terms of timing of surgery

Timing	No. of patients	Total no. of LMRAs	Prevalence
Acute and subacute	383	45	11.7%
Chronic	149	27	18.1%

### Sports activity

There was no correlation between Tegner score group (three groups corresponding to scores of 10–9, 8–5, and < 5) and incidence of LMRA (${\chi ^2}$: 3.287;* P*: n.s). In detail, 122 patients with Tegner scores of 10 and 9, 346 patients with Tegner scores of 8–5, and 64 patients with Tegner scores of < 5 underwent ACL reconstruction. LMRA was detected in 11 patients in the first group, in 53 patients in the second group, and in 8 patients in the third group (Table 5).

**Table 5:** Prevalence of LMRA in terms of Tegner score

Tegner	No. of patients	Total no. of LMRAs	Prevalence
10–9	122	11	9%
8–5	346	53	15.3%
1–4	64	8	13%

### Anterior tibial translation

Preoperative anterior tibial translation, as measured by a KT-2000 arthrometer according to the IKDC classification, was grade B in 254 (47.7%) patients, grade C in 242 (45.4%) patients, and grade D in 36 (6.7%) patients. LMRA was present in 33, 39, and 0 patients, respectively (Table 6). There was no linear correlation between increase in anterior tibial translation and incidence of LMRA (Spearman’s coefficient: − 0.016).

**Table 6:** Prevalence of LMRA in terms of KT-2000 anterior tibial translation

Grade	No. of patients	Total no. of LMRAs	Prevalence
B	254	33	13%
C	242	39	16.1%
D	36	0	0%

### Concomitant meniscal injuries

The prevalence of meniscal tears other than LMRA among all 532 patients included in our study was 39% (*n* = 208) for the medial meniscus and 21.4% (*n* = 114) for the lateral meniscus. 79 patients (14,85%) had a tear in both the lateral and medial menisci.

In the 383 knees with an acute or subacute ACL injury, we found 129 tears (33.6%) of the medial meniscus and 82 (21.4%) of the lateral meniscus. Among these patients with acute or subacute injuries, 31 also had an associated LMRA. An isolated LMRA was present in 14 patients.

In the 149 knees with chronic ACL insufficiency, there were 79 tears (53%) of the medial meniscus and 32 (21.4%) of the lateral meniscus. Among these patients with chronic ACL insufficiency, 16 also had an associated LMRA. An isolated LMRA was present in 11 patients.

The most common morphological types of tear seen for the LMRAs in both groups were a longitudinal tear in the medial meniscus (*n*: 117) and a radial tear in the lateral meniscus (*n*: 45). However, the only significant association detected was with a meniscocapsular tear of the medial posterior horn—a “ramp lesion” (${\chi ^2}$: 7.81;* P*: 0.006).

Table 7 summarizes the specific meniscal injury patterns that were associated with LMRA in our series.

**Table 7:** Associations of lateral meniscal root avulsion with concomitant meniscal lesions detected at the time of ACL reconstruction

Meniscus involved	Type of lesion	No. of acute or subacute injuries	No. of LMRAs associated with acute or subacute injuries	No. of chronic injuries	No. of LMRAs associated with chronic injuries	Total no. of LMRAs	${\chi ^2}$	*P*
Medial meniscus	Longitudinal	75	9	42	6	15	2.36	n.s.
	Flap	19	4	18	1	5	0.55	n.s.
	Radial	7	1	6	2	3	0.19	n.s.
	Ramp	28	5	13	4	9	7.81	0.006
Lateral meniscus	Longitudinal	24	2	0	2	4	0.10	n.s.
	Flap	17	4	8	0	4	0.87	n.s.
	Radial	37	6	8	1	7	1.92	n.s.

No horizontal lesions of the medial or lateral meniscus were seen.

When a ramp lesion (*n* = 41 patients) was present, it was repaired using a posteromedial portal and an all-inside technique, employing an arthroscopic hook with a PDS 0 suture, as described by Morgan [32]. Any other eligible medial or lateral meniscal tear was repaired with all-inside suture devices such as the Smith and Nephew FastFix™, Mitek Truespan™, Arthrex Scorpion™ with Fiberwire 0 suture, or outside-in with a PDS 0 or Fiberwire 0 suture, depending on the site and morphology of the lesion.

## Discussion

**Fig. 1:**
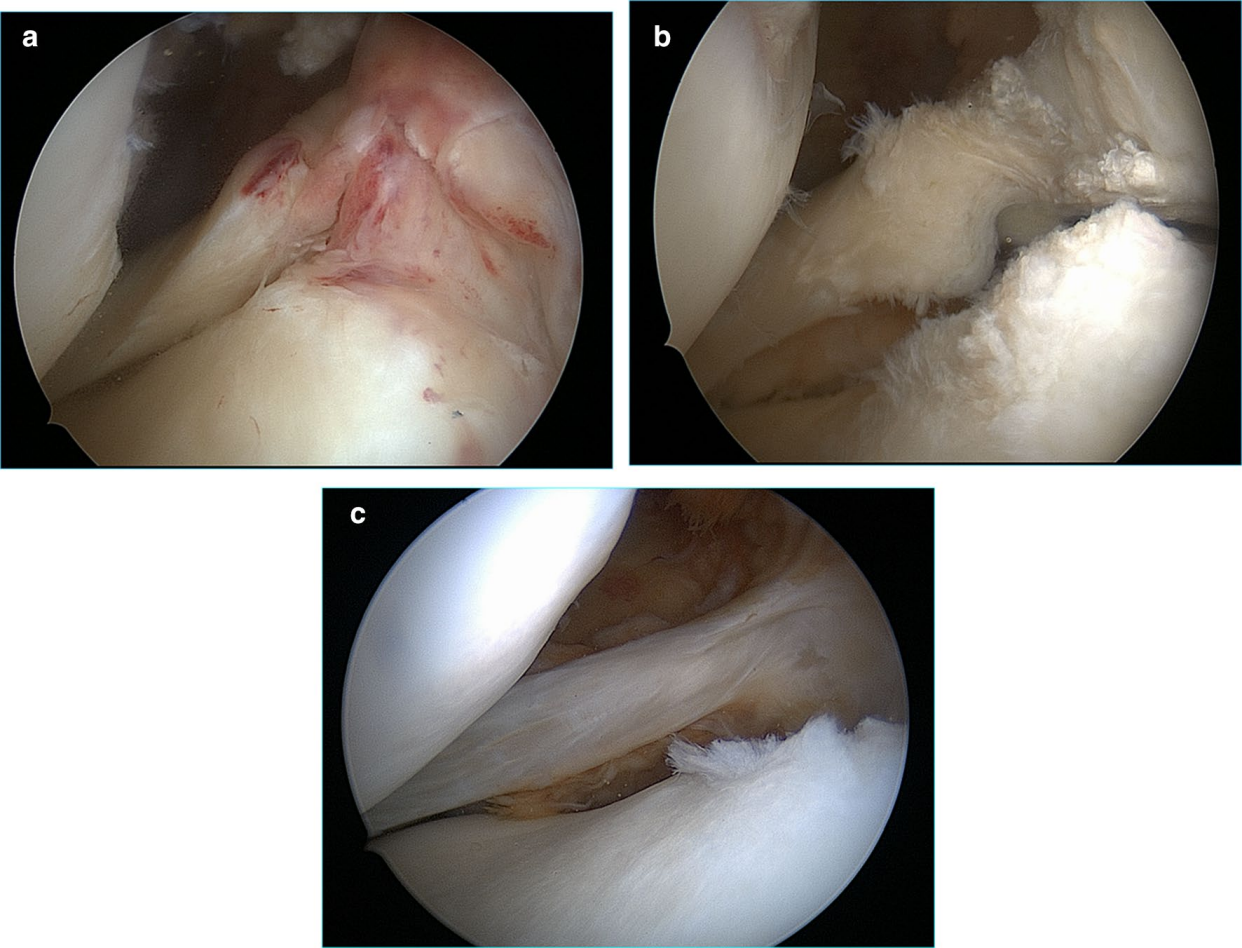
Lateral meniscal root avulsion in the right knee from the anterolateral portal: **a** acute avulsion, **b** chronic avulsion, **c** inner loss type

This study confirmed that LMRA is a relatively common finding in patients with an associated ACL injury, and that its incidence remains high even in chronic cases. The incidence of LMRA in our study, 13.1%, seems to be slightly higher than those previously reported in other papers. Ahn et al. [2] reported an incidence of 6.7%, but this also included radial or longitudinal tears located within 1 cm of the bony insertion. On the basis of arthroscopic findings, they described four types of LMRA: (1) a radial tear with oblique flap, (2) longitudinal cleavage between the bony insertion and MFL insertion, (3) an acute T-type, and (4) a chronic inner loss type. The LMRA incidence observed in our study is quite similar to that reported by Forkel and Petersen [6] for their prospective analysis. They distinguished three types: type 1 is a single avulsion of the root, type 2 is a radial tear close to the root, and type 3 is a complete rupture of the root and the meniscofemoral ligament. Those authors did not give further information on the timing of the ACL tear at surgery, making a comparison with our study difficult. In our arthroscopic evaluations, we categorized avulsions into only three types: acute, chronic, and an inner loss type (Fig. 1a, b, and c, respectively), and we excluded any other lesions that occurred near the meniscal root.

Praz et al. [33] reported a prevalence of 6.6% in a series of 3956 patients undergoing ACL reconstruction. They also reported that there was no statistically significant difference in prevalence between male and female patients, a higher incidence in contact sports, and an association with medial meniscal tears.

Okoroha et al. [34] reported on the incidence of LMRA in relation to tibial alignment in acute ACL ruptures. They reported a LMRA incidence of 10.3% in cases of acute ACL injury among a series of 200 patients treated for ACL rupture—a result closer to ours, considering that they did not include chronic cases in their paper. They also found positive relations between LMRA and greater tibia vara angle, increased tibial slope, and higher BMI. We did not report these data in our paper, and this could be an interesting issue to examine in future studies.

Considering the observed increased incidence of LMRA in chronic cases, one may question whether the avulsion occurred at the time of the ACL injury and did not heal or whether it occurred later, as a result of laxity associated with ACL insufficiency. Untreated radial or longitudinal tears of the posterior horn of the lateral meniscus appear to have a higher chance of healing than medial meniscus tears [10–15] for several reasons: there is better blood supply to the posterior horn of the lateral meniscus in comparison to the pars intermedia [16]; concomitant ACL reconstruction also promotes healing as a result of intraarticular blood clotting [17, 18]; and increased joint stability may reduce forces on the lesion, enhancing the chances of spontaneous healing. Shelbourne and Heinrich [13] reported that stable radial flap tears and peripheral posterior horn tears that did not extend more than 1 cm anterior to the popliteus tendon could be treated successfully by leaving them in situ. Lee et al. [15] performed a second-look arthroscopy and an MRI follow-up evaluation of stable posterior horn tears detected during ACL reconstruction. Their arthroscopic and radiological findings support the idea that stable posterior horn tears of the lateral meniscus may be left in situ to heal spontaneously.

Similar results were reported by Yagishita et al. [18], who noted that 74% of 42 lateral meniscus tears were considered to be healed on second-look arthroscopic surgery. Given the reported evidence in the literature of spontaneous healing of lateral meniscal tears, it is therefore surprising that we detected a high incidence of chronic lesions—perhaps suggesting that a chronic instability could lead to a new LMRA—in our series.

The lateral meniscal root has two distinct insertions: one is anterior and attached to the posterior aspect of the tibial intercondylar eminence, and the other is posterior and confluent with the meniscofemoral ligament [19]. The latter insertion probably prevents spontaneous healing of the bony insertion, if avulsed, as continuous traction by the meniscofemoral ligament makes the root unstable [20]. Also, a proper root avulsion and a radial tear occurring close to the root potentially differ in their healing characteristics and biomechanics. Schillhammer et al. [21] recently demonstrated that detachment of the lateral meniscus posterior horn leads to an increased peak in tibial contact pressure in the lateral compartment, with a decreased average tibial contact area when there is a complete loss of hoop tension. On the contrary, a radial or longitudinal tear of the posterior horn has less of a loading effect on the tibial cartilage because of the integrity of part of the circumferential fibers of the meniscus. Bedi et al. [22] reported that radial tears of up to 60% of the rim width that do not disrupt the continuity of all the circumferential fibers had no significant effect on the peak contact pressure.

To our knowledge, no previously published study of LMRAs has differentiated root avulsions from other types of tears occurring at the posterior horn. There are likely two distinct patterns of tear with different outcomes and consequences, and one must differentiate a true avulsion from a radial or longitudinal tear of the lateral posterior horn. Despite its greater blood supply, an avulsion of the root does not have the same potential for spontaneous healing as tears occurring close to the posterior horn, where continuity of the circumferential fibers is guaranteed.

In contrast to what happens with a medial meniscus root avulsion, there is so far no evidence that LMRA leads to early degeneration of the lateral compartment. While the effects of a radial or root tear in the medial meniscus posterior horn are well known [23–26] (meniscal extrusion and progression to articular cartilage loss, osteoarthritis, or insufficiency fractures—bone marrow edema), in the presence of a chronic LMRA, arthroscopic examination of the lateral compartment often fails to show any significant cartilage lesions or progression to arthritis. The main difference between the medial and lateral compartments is the reduced extrusion of the lateral meniscus compared to that of the medial meniscus. There are two anatomical reasons for this reduced extrusion: the presence of the meniscofemoral ligaments and the popliteus tendon. The meniscofemoral ligaments anchor and protect the lateral meniscus from tearing [20]. When a root tear occurs, lateral meniscus extrusion has been found to be associated with the absence of both meniscofemoral ligaments [4]. The popliteus tendon may then act as a bumper against the meniscal extrusion, or it may have an active role in counteracting the force of the meniscofemoral ligaments, providing coupled control of the mobile posterior arch of the lateral meniscus. The absence of or reduction in lateral meniscus extrusion as compared to medial meniscus extrusion after LMRA may explain the lack of significant adverse effects on the cartilage in the short and long term. Meniscal extrusion is often associated with symptomatic osteoarthritis and joint narrowing [25, 26], so it is not surprising that only mild lateral joint space narrowing is detected when posterior root tears of the lateral meniscus are left in situ, even at a mean of 10 years follow-up [13, 14]. LaPrade et al. described how increased anterior and rotatory instability due to LMRA may contribute to increased loads on an ACL reconstruction graft [29], and evidence of this has been provided by Shybut et al. and DePhillipo et al. [30, 31]. Pula et al. showed that LMRA does not appear to result in meniscal extrusion on MRI at the time of injury [8].

Due to the fact that this particular type of meniscal tear is often asymptomatic or can remain clinically quiescent, it is still unclear whether an acute LMRA requires treatment and what its short- and long-term clinical consequences are [29–31]. LMRAs appear innocuous when observed a long time after the injury, but it is suggested—though not proven—that defunctioning of the lateral meniscus could increase rotational instability of the knee, leading to a failed ACL reconstruction and consequent surgical revision. The clinical consequences of this meniscal defunctioning, in terms of cartilage or further meniscal damage, are yet to be clarified.

Another interesting finding of our study is the significant association of LMRAs with ramp lesions. Liu et al. [27] observed that there is an increase in the prevalence of ramp lesions over time after ACL injury, similar to the increase in LMRA prevalence over time that we detected in the present study. So far, the pathogenesis of each of these lesions is not clear, but we may suppose that both lesion types involve a similar injury mechanism, mainly for acute lesions. One supposed mechanism may be the engagement of menisci during the subluxation of the femoral condyles over the tibia at the time of the ACL tear [28]. The mobility differences between the medial and lateral menisci imply different anatomic functions and therefore different biomechanics in the genesis of lesions. In the cases of acute LMRA in our series, patients often recalled a forceful, rotatory, noncontact mechanism. As shown by Poyton et al. [20], the meniscofemoral ligaments cause medial, superior, and anterior displacement of the lateral posterior horn. A rotational injury may cause forceful traction of the meniscofemoral ligaments, with resultant root avulsion. Brody et al. [4], analyzing LMRAs via MRI, postulated an injury mechanism of valgus external rotation associated with anterior displacement of the tibia, but further studies must be performed to clarify the pathogenesis of this particular injury. As previously reported, anatomical factors could also be risk factors for the mechanical pathogenesis of both LMRAs and ramp lesions, especially an increased posterior tibial slope both medially and laterally and increased asymmetry between the lateral and medial posterior tibial slopes [35].

## Limitations

There are several limitations of this study: (1) the clinical symptoms of LMRAs in both acute and chronic cases were not determined; (2) MRI data for patients were not correlated with the diagnosis of LMRA; (3) in the chronic group, MRI evaluations during the acute phase were not always available, meaning that LMRAs were not diagnosed at the time of initial injury and the traumatic mechanism could not be inferred; (4) patients treated nonoperatively were not included in this study because the diagnosis of LMRA was not confirmed without arthroscopy; and (5) the potential for asymptomatic or clinically quiescent patients with a previous lesion was not considered—only LMRAs found in association with ACL rupture were included in the study.

## Conclusion

LMRA is a relatively common injury associated with an acute or chronic ACL tear. The prevalence of LMRA in our series was more than the prevalences noted in previous reports. Considering this high prevalence, our study highlights the importance of carefully inspecting the lateral meniscal attachment during ACL surgery, especially when a ramp lesion of the medial meniscus is present; otherwise, a lesion that could potentially be responsible for the failure of the ACL reconstruction surgery could be overlooked. The relatively high incidence of chronic ACL tears suggests that lateral root avulsions often do not heal spontaneously, and that these lesions can be differentiated from tears occurring near the root, which have greater potential for spontaneous healing. Moreover, while recent research has shed light on the devastating consequences of medial root avulsion, further studies are necessary to outline the causes and long-term clinical consequences of LMRA.

## Data Availability

All data generated or analyzed during this study are included in this published article.

## Abbreviations

ACL: Anterior cruciate ligament
LMRA: Lateral meniscal root avulsion
